# Effects of Calcium Alginate Submicroparticles on Seed Germination and Seedling Growth of Wheat (*Triticum aestivum* L.)

**DOI:** 10.3390/polym10101154

**Published:** 2018-10-16

**Authors:** Jinxia He, Ruixin Li, Xue Sun, Wenxia Wang, Jianen Hu, Hongguo Xie, Heng Yin

**Affiliations:** 1 Liaoning Provincial Key Laboratory of Carbohydrates, Dalian Institute of Chemical Physics, Chinese Academy of Sciences, Dalian 116023, China; jxhe2016@163.com (J.H.); liruixinlndl@163.com (R.L.); sunxueflies@163.com (X.S.); wangwx@dicp.ac.cn (W.W.); 2 School of Food Science and Engineering, Dalian Ocean University, Dalian 116023, China; 3 School of Environment Science and Engineering, Dalian Maritime University, Dalian 116026, China

**Keywords:** calcium alginate submicroparticles, seed germination, seedling growth, wheat

## Abstract

Calcium alginate (CaAlg) submicroparticles have a potential application in agricultural delivery systems. This study investigated the effects of CaAlg submicroparticles on seed germination and seedling growth of wheat. CaAlg submicroparticles with a Z-average diameter of around 250.4 nm and a measured zeta potential value of about −25.4 mV were prepared and characterized by dynamic light scattering (DLS), scanning electron microscopy (SEM) and energy dispersive X-ray spectrometer (EDS). After this, the effects of the concentration of CaAlg submicroparticles (10–500 μg/mL) on germination percentage, seedling length, the number of adventitious roots, chlorophyll content and soluble protein content were evaluated. The results demonstrated a significant increase in the level of germination percentage (9.0%), seedling index (50.3%), adventitious roots (27.5%), seedling length (17.0%), chlorophyll (8.7%) and soluble protein contents (4.5%) at a concentration of 100 μg/mL. However, an inhibitory effect was observed at a concentration of 500 μg/mL. The SEM examination showed that CaAlg submicroparticles could be successfully adsorbed onto the surface of the wheat seed. Further studies proved that CaAlg submicroparticles at a concentration of 100 μg/mL promoted the expression of indole-3-acetic acid (IAA)-related genes (*YUCCA9*, *AUX1*, *ARF* and *UGT*) in wheat, which resulted in an increase of 69% and 21% in IAA concentration in wheat roots and shoots, respectively.

## 1. Introduction

Alginate, which is a natural acidic linear polysaccharide derived from the cell walls of brown algae, is composed of α-l-guluronate (G) and β-d-mannuronate (M) (uronic acids) [1,2]. The residues are arranged in a block structure of a homopolymer (polyguluronate or polymannuronate) or heteropolymer (a mixed sequence of these residues). Alginate is an inexpensive, biocompatible and environmentally benign biopolymer with numerous applications in the food industry as a non-toxic food additive, thickening agent, gelling agent, emulsifier and colloidal stabilizer [3]. In addition, it was reported that the alginate-treated cotton fabric has antibacterial activity against nosocomial pathogens [4]. Alginate has been widely implicated in wound healing, delivery of bioactive agents and cell transplantation due to its structural similarities to the extracellular matrices of living tissues [5]. This natural polysaccharide is widely used in the medical and food industries. It was also reported that alginate can increase shoot and root lengths, number of leaves, yield and quality, chlorophyll contents, carbonic anhydrase activity, nitrate reductase activity and proline content of crops and plants [6,7].

Recently, biopolymeric nanoparticles and submicroparticles are used in drug delivery systems due to their additional advantages, such as its marine availability (alginate and its derivatives), biodegradability, biocompatibility and nontoxicity [8]. It was reported that alginate nanoaggregates can be used to deliver various materials, such as DNA [9], antisense oligonucleotides [10], antigen [11] and insulin [12]. Submicroparticles have a nanometer size effect and they can enhance the bioactivities [13]. Few studies have applied the polysaccharide submicroparticles in the agricultural field, such as the incorporation of nitrogen, phosphorus and potassium (NPK) fertilizer into the submicroparticles to save fertilizer consumption [14]. Wheat (*Triticum aestivum* L.) is an important food crop. The effective use of agricultural inputs leads to economic benefits and environmentally friendly policies through the minimum input–maximum efficiency approach. To our knowledge, there is no information about the effects of alginate submicroparticles on the growth of wheat.

In this work, we prepared CaAlg submicroparticles by the chemical cross-linking method. The particle size and stability of these submicroparticles were tested by scanning electron microscopy (SEM) and the nanoparticle size analyzer. The effects of CaAlg submicroparticles on seed germination and seedling growth of wheat were investigated. The study found that CaAlg submicroparticles could promote growth at a lower concentration than alginate. Furthermore, CaAlg submicroparticles promote wheat growth by activating the indole-3-acetic acid (IAA) signaling pathway.

## 2. Materials and Methods

### 2.1. Preparation of CaAlg Submicroparticles

Calcium alginate (CaAlg) submicroparticles were prepared based on the ionotropic pre-gelation of polyanion with calcium chloride through a previously described protocol [15]. Briefly, 7.5 mL of 18 mM calcium chloride solution was dropped for 60 min under 800 rpm into a beaker, which contained 120 mL of a 0.06% (*w*/*v*) sodium alginate (NaAlg) solution.

### 2.2. Characterization of CaAlg Submicroparticles

A Zetasizer NanoZS90 (Malvern Instruments, Malvern, UK) was used to determine the zeta potential and particle size of the CaAlg submicroparticles prepared by Dynamic Light Scattering (DLS) at 25 °C, using a Zetasizer NanoZS90 (Malvern Instruments). For each sample, the measurement was repeated three times.

The morphology and nanostructure of CaAlg submicroparticles were characterized by SEM (EOL JSM-7800F, Tokyo, Japan) at an acceleration voltage of 1.0 kV.

### 2.3. Adsorption of CaAlg Submicroparticles on Wheat

Wheat cultivar (*Triticum aestivum* L. Xiaoyan 22) were used in this work. A portion of 30 wheat seeds were placed in 50-mL tubes containing a blank medium and the same medium supplemented with ddH_2_O and CaAlg submicroparticles (500 μg/mL) aqueous solution, respectively. The high dosage was selected due to the detection limit of the elements by scanning electron microscopy and energy dispersive X-ray spectrometer (SEM–EDS). The morphology of the coat of the seeds was characterized by SEM. The weight percentage of elements on the wheat seeds surface was measured by energy-dispersive spectroscopy (Inca EDS system, Oxford Instruments, Oxford, UK).

### 2.4. Wheat Growth Condition

The surfaces of the wheat seeds were sterilized in 75% ethanol for 5 min, before being washed four times with sterile distilled water. The seeds were soaked in a CaAlg submicroparticles aqueous solution (10, 50, 100, 300 and 500 μg/mL) for 8 h, before being germinated in the dark. The plants were transferred into a hydroponic culture at a day/night cycle of 12 h/12 h and temperatures of 25 ± 2 °C/20 ± 2 °C, respectively, with 60% relative humidity. After 7 days, the physiological indexes were evaluated, including germination percentage, germination index, seedling index, seedling length, adventitious root number and roots/shoots ratio. The germination index was calculated as GI = (Gt/Tt), where Gt is the accumulated number of germinated seeds on day t and Tt is the time corresponding to Gt in days [16,17]. The ratio of roots/shoots was counted by (underground fresh weight/aboveground fresh weight). The samples were kept frozen at −80 °C for later biochemical analysis. 

### 2.5. Determination of Chlorophyll and Soluble Protein Content

The chlorophyll (Chl) content in the seedlings of wheat was analyzed according to the method described by Liu [18]. Briefly, 0.5 g of leaves were homogenized in 10 mL of 95% ethanol in mortar. The homogenate was centrifuged at 1500× *g* for 20 min and the supernatant was collected. The absorbance of the collected supernatant was tasted at 665 (A_665_) and 649 (A_649_) nm, respectively. The Chl content in leaves was calculated according to the following formula:

Chl-a = 13.95A_665_ − 6.88A_649_ (1)

Chl-b = 24.96A_649_ − 7.32A_665_ (2)

Chl Content (mg g^−1^ FW) = (Chl-a + Chl-b) × Extraction liquid volume (mL) × Diluted times/Fresh weight (g) (3)

The soluble protein content in the seedlings of wheat was analyzed using the method described by Zhang [19]. Briefly, 0.5 g of leaves were homogenized in 10 mL of 0.1 M phosphate buffer (pH of 7.0) in mortar. The homogenate was centrifuged at 1000× *g* for 10 min and the supernatant was collected. The absorbance of the collected supernatant was tasted at 280 (A_280_) and 260 (A_260_) nm, respectively. The soluble protein content in leaves was calculated according to the following formula:

Soluble protein content (%) = (1.45A_280_ − 0.74A_260_) × Diluted times/Fresh weight (g) (4)

### 2.6. Determination of IAA Content and IAA Oxidase Activity

The IAA content in the seedlings of wheat was analyzed according to the method described by Zhang [20]. Briefly, 1 g of the frozen plant tissue sample was homogenized for 30 min in a chilled mortar with 2 mL of cold 80% (*v*/*v*) methanol containing 1 mM 2,6-di-tert-butyl 4-methylphenol. The homogenate was centrifuged at 10,000× *g* for 20 min at 4 °C. The supernatants were purified using a C18-Sep-Pak column (Waters, Milford, MA, USA). The chromatographic separation was carried out on an Agilent High-Performance Liquid Chromatography (HPLC), which was equipped with a BDS HYPERSIL C18 (Thermo, Waltham, MA, USA, 250 nm × 4.8 mm, 5 μm) column. The mobile phase was composed of methanol (phase B) and acetate buffer (phase A) (pH was adjusted to 3.22 by acetic acid), while the proportion of mobile phase was 45% (A): 55% (B) and the flow rate was 0.8 mL/min. The detection wavelength was set at 280 nm and the injection volume was 20 μL. The concentration of IAA was calculated based on the peak area. 

The IAA oxidase activity in the shoots and roots of wheat were analyzed according to the method described by Gibson and Zhang [20,21]. Briefly, 1 g of the frozen plant tissue sample was homogenized in a chilled mortar with 6 mL of cold 0.02 M phosphate buffer (pH of 6.0). The homogenate was centrifuged at 1500× *g* at 4 °C for 20 min. The supernatant was used for assaying the activity of IAA oxidase. The reaction mixture (samples of 10 mL, each containing 2 mL of 200 g/mL IAA, 1 mL of 1 mM MnCl_2_, 1 mL of 1 mM 2,4-dichlorophenol, 5 mL of 0.02 M phosphate buffer and 1 mL of enzyme solution) was incubated at 25 °C for 30 min. A total of 2 mL of the reaction solution was colored by adding 4 mL of FeCl_3_–H_2_SO_4_–H_2_O (3–60–100) solution at 40 °C for 30 min, before the absorbance was measured at 530 nm.

### 2.7. Gene Expression Analysis of the Wheat by RT-qPCR

The total RNA of shoot and root were extracted using TRIZOL reagent (Invitrogen, Carlsbad, CA, USA) according to the manufacturer’s instructions. The quantification of RNA was conducted using ScanDrop100 (AnalytikJenaAG, Jena, German). RNA was reverse transcribed into cDNA with AMV Reverse Transcriptase (Takara). Serial dilutions of cDNA were used as the templates in RT-qPCR (qTOWER 2.2, AnalytikJenaAG, Jena, German) using a SYBR Green kit (Bio-Rad, Hercules, CA, USA) with gene-specific primers designed using Premier 6.0 (Table 1). Raw data were analyzed using ddCt methods of Livak and Takato [22,23] and were all normalized to the expression of β-tubulin in each group.

### 2.8. Statistical Analysis

The differences among treatments were analyzed, taking *p* < 0.05 as the significance level, according to Duncan’s multiple range test. Statistical procedures were carried out with the software package SPSS11.0. Each experiment was repeated three times under the same conditions in the work.

## 3. Results

### 3.1. Characterization of CaAlg Submicroparticles

**Figure 1:**
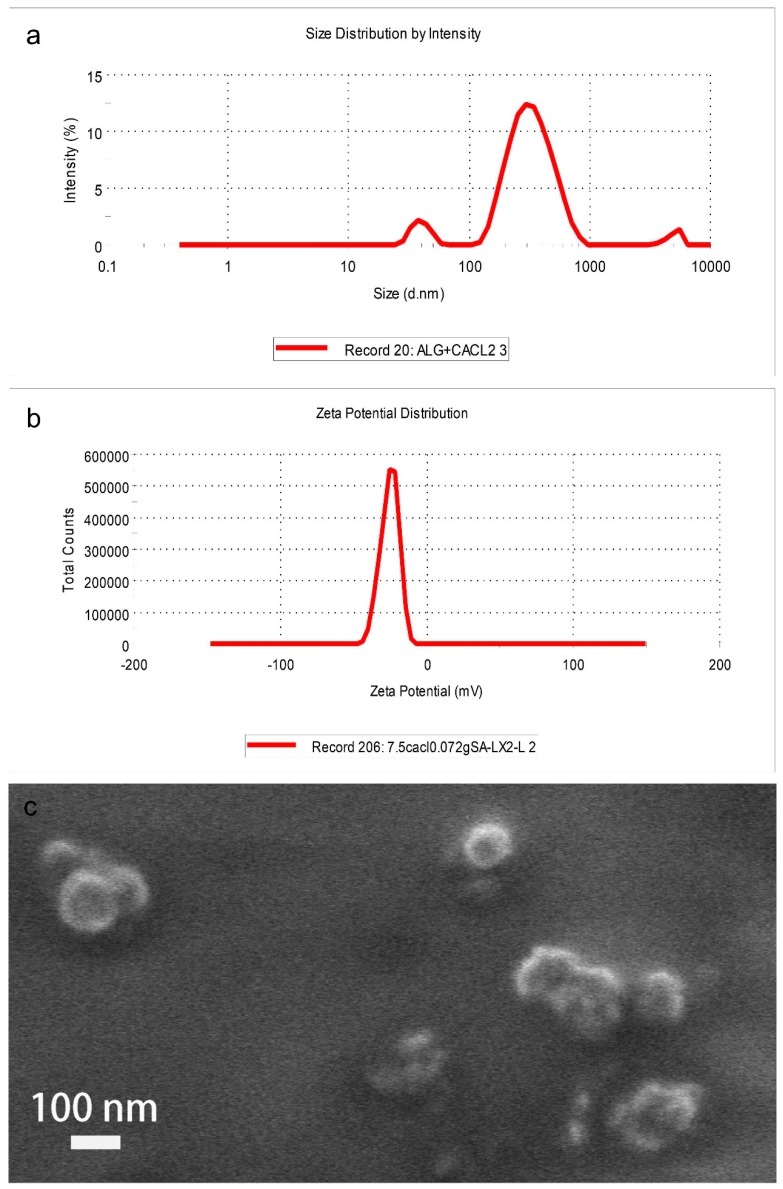
The size distribution of CaAlg submicroparticles (**a**), the zeta of CaAlg submicroparticles (**b**) and the scanning electron microscopy (SEM) image of CaAlg submicroparticles (**c**).

The results of DLS were shown in Figure 1. The Z-average of the CaAlg submicroparticles (Figure 1a) was 250.4 nm, with a polydispersity index (PDI) of 0.42. A PDI value < 0.5 is good for colloidal suspension. It portrays that the suspension has homogenous size distribution. Besides, the CaAlg submicroparticles synthesized in this work had a zeta potential value of −25.4 mV. The results indicated that the CaAlg submicroparticles had good stability (Figure 1b) as a zeta potential value that is around ±30 mV is assumed to be good for suspension. The typical morphology of the CaAlg submicroparticles examined with SEM is shown in Figure 1c. The CaAlg submicroparticles exhibited a spherical shape with diameter range of 80–120 nm. The particles were well dispersed. It is common that the particle size given by SEM analysis is smaller than that by DLS, which may be due to different principles involved in these two techniques. CaAlg submicroparticles are measured in a dry state in SEM analysis while the measurement involves the hydrodynamic state of submicroparticles in DLS, which creates more DLS.

### 3.2. Experiment of CaAlg Submicroparticles Adsorption of Wheat Seeds

**Figure 2:**
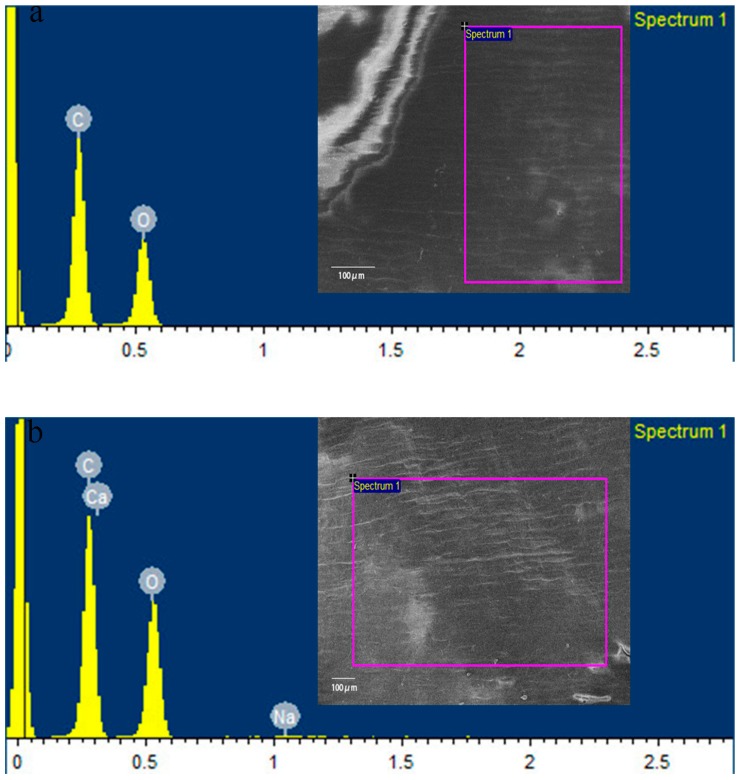
Scanning electron microscopy and energy dispersive X-ray spectrometer (SEM–EDS) of wheat seeds soaked in ddH_2_O (**a**) and CaAlg submicroparticles (**b**).

EDS in conjunction with SEM was used to determine the elemental composition of the wheat pericarp soaked in ddH_2_O and in the solution containing CaAlg submicroparticles. As a representative of all the samples, the data for seeds soaked in ddH_2_O or CaAlg submicroparticles are presented in Table 2 and Figure 2. 

As seen in Table 2 and Figure 2, the seeds soaked in CaAlg submicroparticles have a higher weight percentage of calcium and sodium on the surface compared to seeds soaked in ddH_2_O. Generally, Ca comes directly from the crosslinking reagent. A small amount of Na indicated that a small fraction of NaAlg did not bind with calcium ions. The results demonstrated that CaAlg submicroparticles could be adsorbed onto the surface of wheat seeds.

### 3.3. Effects of CaAlg Submicroparticles on Seed Germination and Plant Biomass

**Figure 3:**
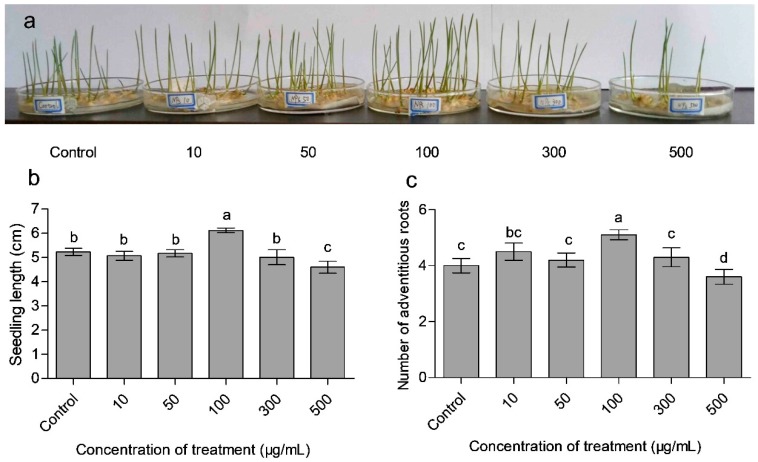
Effects of CaAlg submicroparticles on phenotypes (**a**), seedling length (**b**) and adventitious roots (**c**) of wheat. Each value represents the mean ± SE (standard error) (*n* = 10). Different lowercase letters indicate significant differences at *p* < 0.05.

To ascertain the growth promoting effects of CaAlg submicroparticles on wheat seedlings, the growth characteristics, such as phenotype (Figure 3a), seedling length (Figure 3b), the number of adventitious roots (Figure 3c) and physiology index (Table 3), were evaluated. After applying 100 μg/mL CaAlg submicroparticles to the wheat, the germination percentage increased by 9.0%, the seedling index increased by 50.3%, the number of adventitious roots increased by 27.5% and the length of seedlings increased by 17.0%. However, the results showed that the above-mentioned growth characteristics worsened when the CaAlg submicroparticles were added at a concentration of 500 μg/mL. It was established that the plant oligosaccharides and polysaccharides exhibited positive physiological activity in the range of low concentrations and showed inhibitory activity at high concentrations [24,25]. The results indicated that the applied concentration of submicroparticles or polysaccharides with physiological activity should be adjusted in order to obtain the optimal concentration [26].

### 3.4. Effects of CaAlg Submicroparticles on Chorophyll Content of Wheat

**Figure 4:**
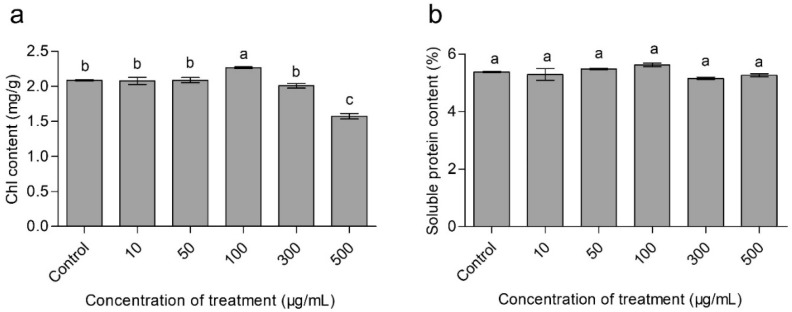
Effects of CaAlg submicroparticles on chlorophyll (**a**) and soluble protein content (**b**) of wheat. Each value represents the mean ± SE (*n* = 3). Different lowercase letters indicate significant differences at *p* < 0.05.

The chlorophyll content is considered as an index of the total amount of light harvesting complex and the electron transport components [27], while the relative chlorophyll content is positively correlated with the photosynthetic rate [28]. Figure 4a showed the chlorophyll content in the leaves of wheat plants exposed to various concentrations of CaAlg submicroparticles. The results showed that the levels of chlorophyll increased by 8.7% in the 100 μg/mL CaAlg submicroparticles treatment group compared to the control group.

### 3.5. Effects of CaAlg Submicroparticles on Soluble Protein Content of Wheat

The total soluble protein content is an important physiological parameter for plant physiological metabolism, which reflects protein synthesis, degradation and metabolism processes [29]. Increasing the photosynthetic pigment contents will boost the plant growth rate and biomass, which will eventually enhance the level of protein, starch contents and yield components in crop plants [30,31]. It was also reported that soluble proteins can protect plant cells from potential oxidative damage, which is caused by the deposition of nanoparticles on plants [32]. Figure 4b showed the level of total soluble protein contents in the leaves of wheat plants treated with different doses of CaAlg submicroparticles. CaAlg submicroparticles at a concentration of 100 μg/mL enhanced the total protein contents by 4.5%, compared to the control group. The results were similar to previous studies.

### 3.6. Effects of CaAlg Submicroparticles on IAA Synthesis and Metabolism of Wheat

**Figure 5:**
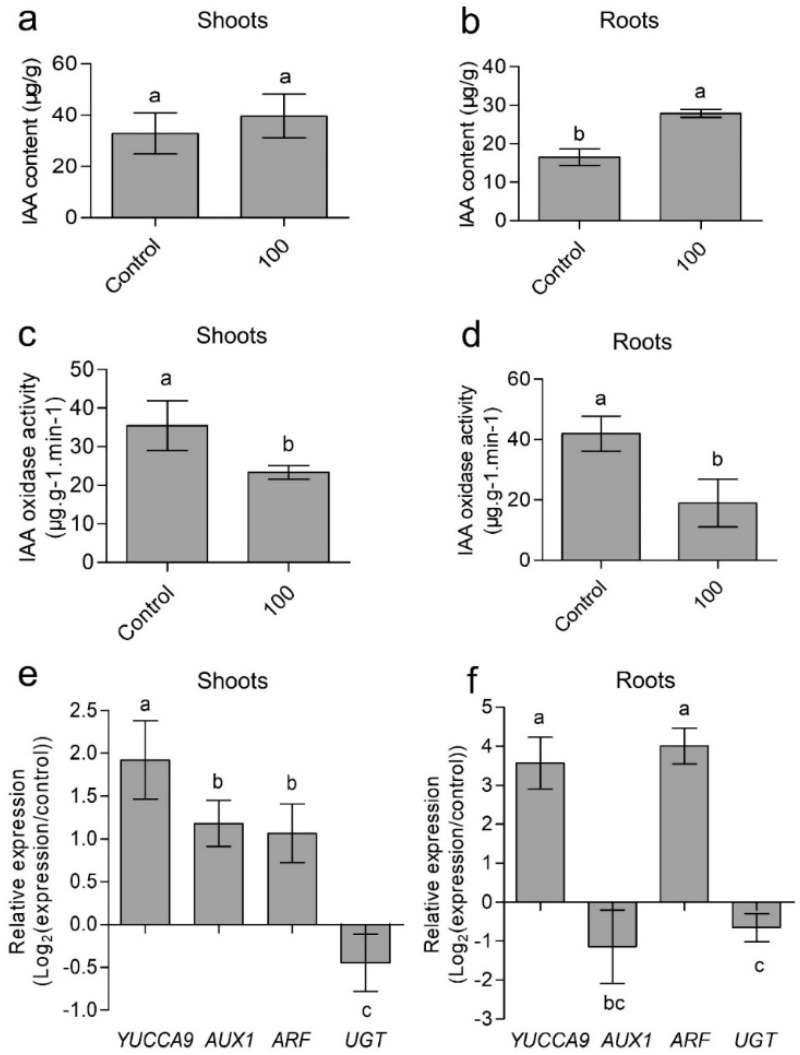
Effects of CaAlg submicroparticles on indole-3-acetic acid (IAA) content (**a**,**b**) and IAA oxidase activity (**c**,**d**) of wheat. Relative expression of *YUCCA9, AUX1*, *ARF* and *UGT* in wheat plants (**e**,**f**). Expression of *YUCCA9*, *AUX1*, *ARF* and *UGT* genes was analyzed using RT-qPCR (qTOWER 2.2, AnalytikJenaAG, Jena, German). Gene expression in response to the submicroparticles treatment is presented on a scale of log_2_ as values relative to those of the control expression levels (defined as 0 on a log_2_ scale). Each value represents the mean ± SE (*n* = 3). Different lowercase letters indicate significant differences at *p* < 0.05. Control: plants grow without submicroparticles; 100: plants were soaked with 100 μg/mL CaAlg submicroparticles.

Indole-3-acetic acid (IAA) is an important plant hormone that is essential for many aspects of plant growth and development. To explore the mechanism of CaAlg submicroparticles regulating wheat development, IAA content and gene expression related to IAA synthesis and metabolism were characterized. As 100 μg/mL CaAlg submicroparticles have the most significant effect on wheat growth promotion, we selected the 100 μg/mL CaAlg submicroparticles treatment group for the subsequent experimental study model. The seedlings were harvested, before the endogenous IAA levels were analyzed in shoots and roots. The endogenous IAA levels were 21% and 69% higher in shoots and roots of CaAlg submicroparticles-treated wheat than in the control shoots and roots (Figure 5a,b), respectively.

To further explore whether or not the increase in IAA found in the CaAlg submicroparticles group is related to gene expression involved in IAA synthesis, transcription and metabolism, we determined *YUCCA9*, *AUX1*, *ARF* and *UGT* gene expression. YUCCA, which encodes flavin monooxygenase-like proteins, catalyzes the transformation of Trp to IPyA. YUCCA9 play an important role in IAA signaling pathways [33,34]. In our study, the transcription levels of *YUCCA9* in shoots and roots of CaAlg submicroparticles group increased by 192% and 357% compared to controls (Figure 5e,f). 

AUX1 is an auxin cellular influx carrier [35,36]. In the shoots, 100 μg/mL CaAlg submicroparticles resulted in an increase of 118% in the gene expression of *AUX1* compared to controls (Figure 5e). However, the *AUX1* gene expression in root showed a slight decrease without a significant difference in the CaAlg submicroparticles group in comparison with the control group (Figure 5f). IAA distribution is asymmetric. There provides evidence for the transport of IAA from the shoot to the root system. Furthermore, it is speculated that the upregulation in the *AUX1* gene in shoots may be related to the distribution of IAA in roots and shoots. These results implied that CaAlg submicroparticles probably increased the IAA content by influencing the IAA synthesis and transport.

Furthermore, we analyzed the gene expression of *AUXIN RESPONSE FACTOR* (*ARF*), which is a positive transcriptional regulator [37]. *ARF* increased by 106% and 400% in the shoot and root compared to controls (Figure 5e,f). The results indicated that CaAlg submicroparticles can active the transcription of downstream genes of IAA signal pathway.

Finally, we examined the activity of IAA oxidase and the gene expression of uridine diphosphate glucosyltransferase (*UGT*) [38,39,40]. The activity of IAA oxidase in shoots and roots decreased by 45% and 66% (Figure 5c,d). The transcription levels of *UGT* in shoots and roots also decreased (Figure 5e,f). These results further indicated that the submicroparticles promote seed germination and seedling growth in wheats in a manner that is dependent on the IAA signaling pathway.

## 4. Discussion

The studies confirmed that marine algae polysaccharides and derived oligosaccharides can promote growth in different plants [20,41,42]. The concentration is important for its resulting effect. It was reported that algae polysaccharides at a concentration of 500 μg/mL can stimulate the elongation of carrot and rice roots [7]. The study showed that alginate at a concentration of 2 × 10^4^ μg/mL can promote seed germination and plant length of sunflower [43]. Moreover, it was also reported that alginate-derived oligosaccharides at a concentration of 2000 μg/mL can promote the plant growth of cucumber and tomato [44,45]. In addition, alginate-derived oligosaccharides at concentrations of 750 μg/mL and 1500 μg/mL enhanced germination via the promotion of the amylase activity and acceleration of the metabolic activities of the maize seed [46]. These researches data indicated that alginate and alginate-derived oligosaccharides exert its biological functions at higher concentrations. Furthermore, submicroparticles have a nanometer size effect, which can enhance its bioactivities [13]. The effective use of agricultural inputs leads to economic benefits and environmentally friendly policies through the minimum input–maximum efficiency approach. 

In this paper, the effect of the concentration of CaAlg submicroparticles (10–500 μg/mL) on germination percentage, seedling length, the number of adventitious roots, chlorophyll content and soluble protein content were evaluated. Our study indicated that adding CaAlg submicroparticles at concentrations of 10, 50 and 300 μg/mL has little effect on wheat seed germination. The results demonstrated a significant increase in the level of germination percentage (9.0%), seedling index (50.3%), adventitious roots (27.5%), seedling length (17.0%), chlorophyll (8.7%) and soluble protein contents (4.5%) at a concentration of 100 μg/mL. Our study has showed that CaAlg submicroparticles can exert biological activity at lower concentrations (100 μg/mL). These special properties of submicroparticles can improve the absorption and utilization in plant [47]. 

However, there was a significant inhibition in the level of germination percentage (44.1%), seedling index (32.8%), and seedling length (20.8%) when the submicroparticles were added at a concentration of 500 μg/mL. It was previously reported that the high concentrations of oligosaccharides can induce cell apoptosis in plants [48,49]. We speculated that the high concentrations of CaAlg submicroparticles may have a similar effect on plants.

## 5. Conclusions

The present study investigated the effects of CaAlg submicroparticles on seed germination and seedling growth of wheat. The results showed that 100 μg/mL CaAlg submicroparticles had the greatest effect on wheat growth compared to the 10, 50 and 300 μg/mL treatment groups. At a concentration of 100 μg/mL, CaAlg submicroparticles significantly enhanced the seed germination percentage, germination index, seedling length, the number of adventitious roots, level of photosynthetic pigments and soluble protein contents. The study proved that CaAlg submicroparticles at the concentration of 100 μg/mL promoted the expression of IAA-related genes (*YUCCA9*, *AUX1*, *ARF* and *UGT*) in wheat, which resulted in an increase of 69% and 21% in the IAA concentration in wheat roots and shoots, respectively. These results implied that CaAlg submicroparticles may promote wheat growth by influencing IAA synthesis and metabolism. This study can provide a theoretical basis for the application of CaAlg submicroparticles in agriculture production. However, the complex molecular mechanism during seed germination that was triggered by CaAlg submicroparticles remains unknown. Subsequent studies need to investigate how the CaAlg submicroparticles can induce plant growth via extracellular recognition or entry into cells. Our results also showed that CaAlg submicroparticles increased the utilization of alginate in plants. After this, the mechanism of the nanometer size effect of submicroparticles should be included in subsequent future research, which is beneficial in the application of alginate.

## Figures and Tables

**Table 1:** Polymerase chain reaction primers used in the study.

Gene	Primer Sequence (Forward 5′–3′)	Primer Sequence (Reverse 5′–3′)
*β-tubulin*	CATGCTATCCCTCGTCTCGACCT	CGCACTTCATGATGGAGTTGTAT
*YUCCA9*	GATTCTGGGCATCTCAACA	GGGACCACCTTTATTTCG
*AUX1*	CCGTCATTCCACAACTACC	ACATCGCGTGCATTATCT
*ARF*	AGCCAAAAGCAGAACTACC	AGCCATCCCAAGCACTAT
*UGT*	CTCACCTTTAGTGGCTTTTG	CTCACCATCGCATCTCAG

**Table 2:** Elements on the surface of wheat seeds soaked in ddH_2_O and CaAlg submicroparticles.

Elements	ddH_2_O (%)	CaAlg Submicroparticles (%)
C	57.41	50.06
O	42.59	49.12
Na	-	0.15
Ca	-	0.67
Total	100	100

**Table 3:** Effects of CaAlg submicroparticles on germination of wheat seeds ^1^

Treatment	Fresh Weight (mg explant ^−1^)		Root/Shoot	Seedling	Germination Percentage	Germination
(μg/mL)	Aboveground Part	Underground Part	Ratio	Index	(%)	Index
Control	35.78 b	21.16 b	0.591 bc	33.67 c	74.44 b	25.10 b
10	35.54 b	21.98 b	0.618 b	35.57 b	63.33 c	20.83 d
50	35.29 b	21.15 b	0.599 bc	33.83 a	72.22 b	23.05 c
100	42.36 a	29.73 a	0.702 a	50.60 a	81.11 a	27.02 a
300	35.53 b	21.89 b	0.616 b	35.38 b	63.33 c	21.40 d
500	33.39 c	19.36 c	0.580 c	30.59 d	54.44 d	16.86 e

^1^ Mean values (*n* = 3) with different letters are significantly different (*p* < 0.05), which was calculated by Duncan’s multiple range test.
